# Dissociation Dynamics of Protonated Nitroimidazoles: The Ortho Effect as the Mechanistic Trigger for Radical Formation

**DOI:** 10.1021/acs.jpca.6c04617

**Published:** 2026-10-22

**Authors:** Jorge H. C. Basilio, Ricardo R. Oliveira, Fábio C. S. Nogueira, Nina D. Ortiz, Gilberto B. Domont, Alexander Dorn, Wania Wolff

**Affiliations:** † Physics Institute, 133645 Federal University of Rio de Janeiro, Rio de Janeiro, Rio de Janeiro 21941-909, Brazil; ‡ Chemistry Institute, 28125 Federal University of Rio de Janeiro, Rio de Janeiro, Rio de Janeiro 21941-909, Brazil; § 28297 Max Planck Institute for Nuclear Physics, Saupfercheckweg 1, Heidelberg 69117, Germany

## Abstract

Substituted 5-nitroimidazoles are recognized as being significantly more effective in treating hypoxic tumors than their 4-nitroimidazole isomers. While classical Quantitative Structure–Activity Relationship (QSAR) models point to electron affinity (EA) as the critical parameter for this activity, recent gas-phase electron-attachment studies reveal a paradox: isolated radical anions of 1-methyl-5-nitroimidazole (1M5NI) do not spontaneously fragment into essential cytotoxic species such as NO^•^ and OH^•^ radicals. To resolve this dilemma, we investigate the high-energy dissociation pathways triggered instead of activated during radiotherapy. Because these radiosensitizers capture secondary electrons and are immediately protonated in the acidic tumor microenvironment, we utilized Electrospray Ionization (ESI) Orbitrap mass spectrometry to generate the [M + H]^+^ species. This protonated closed-shell cation serves as a rigorous structural and mechanistic analogue to map the reaction topology of the transient in vivo state. By comparing the protonated 1M5NI ([1M5NI + H]^+^) and 2-methyl-4-nitroimidazole ([2M4NI + H]^+^) isomers through high-level computational modeling [DFT and CCSD­(T)], we elucidate that while EA serves as the initial trigger, the protonated state is mandatory to unlock the fragmentation pathway. Crucially, the massive energy deposition inherent to radiotherapeutic environments provides the necessary driving force to overcome the high-energy dissociation barriers (>3 eV), channeling the internal energy along a specific normal mode of vibration to eject the cytotoxic radicals. These findings provide a fundamental physical chemistry basis for the clinical efficacy of 5-nitroimidazole drugs.

## Graphical abstract

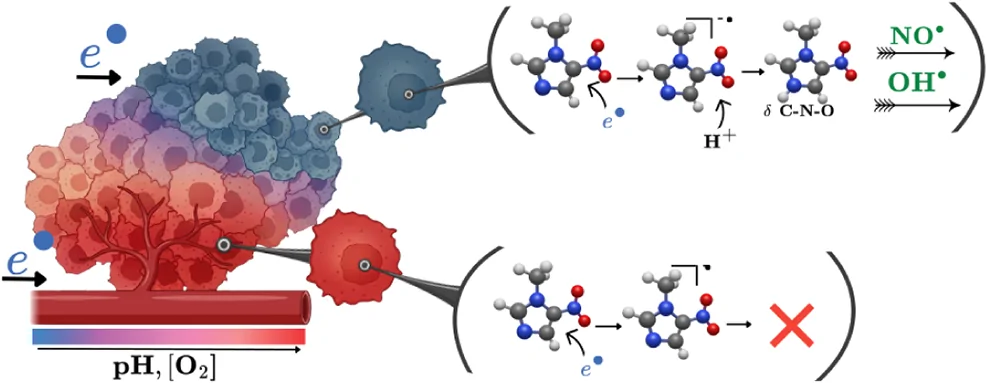

## Introduction

Solid tumors frequently develop regions of severe hypoxia and extracellular acidosis due to rapid cellular proliferation that outstrips their vascular supply and alters cellular metabolism. This low-oxygen environment makes the tissue highly resistant to conventional radiotherapy.^1,2^ To overcome this, nitroimidazole derivatives comprise chemical sensitizers designed to improve the lethal effects of ionizing radiation on tumor cells, particularly within these oxygen-depleted environments. Radiosensitizers permanently fix the DNA damage induced by ionizing radiation (preventing cellular repair mechanisms), mimicking the effects of oxygen in hypoxic tumors.^3,4^ Due to these physicochemical properties, nitroimidazole derivatives continue to be explored for therapeutically modulating the hypoxic tumor microenvironment.^1,5^

The isomer 1-methyl-5-nitroimidazole (1M5NI) (Figure 1) is less stable than 2-methyl-4-nitroimidazole (2M4NI) (Figure 1) due to steric repulsion between the methyl group and the adjacent nitro group. In the isomeric configuration of 1M5NI, the methyl group at position 1 and the nitro group at position 5 are very close, unlike 2M4NI, where the nitro group at position 4 remains distant. This steric hindrance destabilizes the structure of 1M5NI. However, these same factors that make 1M5NI less stable are what make it more reactive than 2M4NI. This is because 1M5NI is capable of reacting internally via the ortho effect, which occurs due to the vicinal methyl group.^6–8^ This structure–reactivity relationship aligns with foundational studies, demonstrating that 5-nitroimidazole scaffolds possess a consistently higher one-electron reduction potential compared to their 4-nitroimidazole analogues.^9–11^ For instance, comprehensive compilations show that 4-nitroimidazoles exhibit highly negative reduction potentials (e.g., the one-electron reduction potential at pH 7, $E_{7}^{1}=-542\;\mathrm{mV}$ for 2M4NI, and ≈−520 mV for substituted derivatives), whereas analogous 5-nitroimidazole derivatives present potentials shifted by approximately 70 to 80 mV toward more positive values (e.g., $E_{7}^{1}\approx -460\;\mathrm{mV}$ ).^11^

**Figure 1:**
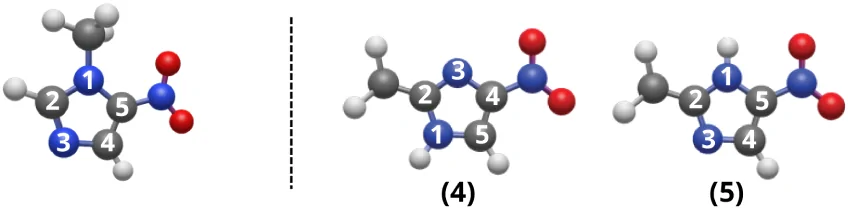
1M5NI (left) and 2M4NI, 2M5NI (right). The counting is done in such a way that the first atom is the substituted nitrogen, and the sequence follows the path that provides the lowest number for the unsubstituted nitrogen of the imidazole ring. Carbon atoms are gray, nitrogen atoms are blue, oxygen atoms are red, and hydrogen atoms are white.

Building upon previous electron ionization (EI) time-of-flight (TOF) mass spectra^12–15^ in this work, we unveil the high-energy gas-phase reactivity of these isomers using high-resolution mass spectrometry (HRMS) coupled with electrospray ionization (ESI). While traditional approaches often seek to study the isolated radical anion, these species typically resist spontaneous fragmentation in the gas phase.^16^ However, our hypothesis is that during clinical radiotherapy, target molecules capture due to their electron affinity secondary electrons and, acting as strong bases, are immediately protonated by the acidic microenvironment characteristic of tumor hypoxia.^11,17–19^ To accurately model this, ESI was used to transfer the intact analytes from an acidic solution directly into the gas phase, generating the [M + H]^+^ closed-shell cation. We employ this protonated state as a rigorous structural and mechanistic analogue to map the reaction topology of the transient, activated in vivo state. Crucially, the massive energy deposition inherent to radiotherapeutic environments provides the driving force required to overcome high-energy dissociation barriers that would be inaccessible under thermal biological conditions. Driven by this energy, the structural configuration of [1M5NI + H]^+^ enables a specific dissociation pathway, predominantly yielding the [M – NO]^+^ and [M – NO – OH]^+^ fragments, which demonstrate its high radical reactivity and high radiosensitizing potential. Conversely, the structural configuration of [2M4NI + H]^+^ favors ring opening and subsequent secondary fragmentation. The formed ions from [1M5NI + H]^+^ describe the loss of NO^•^ and OH^•^ radicals, which are well known and extensively studied in the treatment of tumors, particularly in combating hypoxia-mediated resistance.^20–22^ By employing quantum chemical computations, we modeled the high-energy reaction pathways that explain how [1M5NI + H]^+^ releases the neutral radicals essential to its radiosensitizing activity. Furthermore, we elucidated the competing pathways that drive [2M4NI + H]^+^ to fragment into charged ions with low radiosensitizing effectiveness.

## Methods

### Experimental Section

High-resolution mass spectrometry experiments were conducted on a Q Exactive Plus instrument (Thermo Scientific)^23^ equipped with an Atmospheric Pressure Ionization (API) source configured for Electrospray Ionization (ESI). All analyses were performed using both source-derived ionization (Full MS) and fragmentation via the Higher Energy Collisional Dissociation (HCD) cell.^24^ The samples were introduced into the ionization source by direct infusion, with the ion transfer capillary temperature maintained at 320 °C to ensure efficient droplet desolvation without inducing thermal degradation.^25^

Both analytes, 1M5NI and 2M4NI, were initially dissolved in HPLC-grade methanol at 0.6 mg/mL (1.2 mg in 2 mL) and 1.1 mg/mL (1.1 mg in 1 mL), respectively, to prepare stock solutions. The difference in stock concentrations was required to accommodate the limited solubility of the sterically hindered 1M5NI isomer. Subsequently, 10 μL aliquots of these stock solutions were diluted into 990 μL of an aqueous 0.1% formic acid solution. This solvent system is widely validated to promote efficient protonation and lower the surface tension of the electrospray droplets.^26^ All data reported herein were acquired in positive ion mode (+ESI) with a spray voltage of 3.9 kV.

To comprehensively map the fragmentation pathways, experiments were conducted under four distinct activation regimes: IS-CID alone at 20 and 60 V, and sequential activation using IS-CID (20 or 60 V) followed by HCD at a Normalized Collision Energy (NCE) of 35. The applied IS-CID DC voltages correspond to laboratory-frame energies of 20 and 60 eV for singly charged ions. These voltages accelerate the precursor ions into the transition region, inducing fragmentation through collisions with the residual nitrogen bath gas (local pressure ∼1–2 mbar). In the HCD cell, the nitrogen collision gas pressure is maintained by the instrument’s high-vacuum regime at around 10^–4^ mbar.^27^ For the Thermo Q Exactive system, the absolute laboratory-frame collision energy scales linearly with the precursor *m*/*z*.^28^ At NCE 35, the corresponding absolute laboratory collision energy is approximately 9.0 eV for our precursor ions. It is crucial to emphasize that both the in-source collision-induced dissociation (IS-CID) and HCD regions operate under a multiple-collision regime with the nitrogen bath gas.^24,27^ Consequently, the energetic activation of the ions occurs via sequential, stepwise collisional energy accumulation rather than under a single-collision regime (see kinematic calculations section in the Supporting Information).

### Computational Details

All electronic structure calculations were performed in the gas phase using the ORCA 6.0 program package^29,30^ focusing on the protonated isomers [M + H]^+^ detected under positive-ion electrospray ionization (ESI­(+)) conditions. Modeling this protonated state is highly relevant from a biological perspective because hypoxic tumor microenvironments are distinctly acidic and naturally drive these isomers, upon electron capture during radiotherapy, into the same [M + H]^+^ state observed in our ESI­(+) experiments. Geometry optimizations and harmonic frequency calculations were conducted at the DFT level using the range-separated hybrid functional with dispersion correction, ωB97X-D4 ^31–34^ combined with the def2-TZVP basis set.^35^ Very tight convergence thresholds for geometry (*VeryTightOpt*) and self-consistent field (*ExtremeSCF*) were applied to ensure very precise optimized coordinates. The nature of stationary points as local minima was confirmed by the absence of imaginary frequencies.^36^ Anharmonic corrections were applied using the second-order vibrational perturbation theory (VPT2)^37–39^ at the same level of theory.

For the anharmonic spectra of [1M5NI+H]^+^, a correction was required for two harmonic modes due to a Fermi resonance, where the first overtone of the mode with 716 cm^–1^ coincided with the fundamental with 1432 cm^–1^ (Table S1). Since standard VPT2 is a nondegenerate formalism, it inherently fails to correctly treat such strong couplings, resulting in mathematical singularities and spurious frequency shifts.^40^ To rectify this without resorting to empirical external factors, we derived an intrinsic scaling factor (λ = 0.988). This localized factor is based on the average ratio between the purely harmonic and the accurately calculated anharmonic frequencies in the resonance-free region (<2000 cm^–1^), following the well-established principle of linear frequency scaling.^41^ Applying this intrinsic factor to the coupled modes properly adjusted their frequencies to 707 cm^–1^ and 1414 cm^–1^, respectively, restoring physical consistency to the spectrum.

Reaction pathways were obtained using the Nudged Elastic Band (NEB)^42^ method and relaxed surface scan computations.^43^ The scan searches were applied when the potential energy surface (PES) indicated barrierless dissociation coordinates or to generate initial guess structures for NEB calculations. In simple cases, the energy profiles obtained via relaxed scans were sufficient to describe the reaction pathway. The NEB method was used for complex mechanisms. Specifically, the NEB search was applied to compute the PES, followed by the optimization of the highest energy point as a transition state (TS),^44^ which was confirmed by the presence of a normal mode with a single imaginary frequency.^36^ Both NEB and scan calculations were performed at the same theoretical level as geometry optimizations. For the radiosensitive isomer ([1M5NI+H]^+^), electronic energies were refined using single-point calculations at the CCSD­(T)/cc-pVTZ//ωB97X-D4/def2-TZVP level of theory along the PES.

Population and atomic dipole moment-corrected Hirshfeld (ADCH) partial charge analyses, including the mapping of the molecular electrostatic potential (MESP) surfaces, were carried out using the Multiwfn software.^45^

## Results and Discussion

The experimental results obtained by ESI­(+) (Figure 2) with an applied IS-CID voltage of 20 V (corresponding to a laboratory-frame energy of 20 eV) reveal distinct fragmentation patterns for the two isomers. While [2M4(5)­NI+H]^+^ exclusively exhibits the protonated molecular ion with *m*/*z* = 128.045, [1M5NI+H]^+^ displays two diagnostic fragmentation channels with *m*/*z* = 98.047 and *m*/*z* = 81.045. The molecular ions are protonated due to the addition of 0.1% formic acid, as described in the Experimental Section. The high mass accuracy of the Orbitrap analyzer (<5 ppm)^46^ allows for precise identification and unambiguous assignment of these peaks. The peaks with *m*/*z* = 98.047 and *m*/*z* = 81.045 correspond to the sequential loss of a neutral NO^•^ radical followed by the elimination of an OH^•^ radical, consistent with the formation of the species [1M5NI+H—NO]^•+^ (*m*/*z* = 98.047) and [1M5NI+H—NO—OH]^•+^ (*m/z =* 81.045), respectively. The presence of ions with smaller masses in this region is attributed to common chemical background noise, which is ever-present in high-resolution mass spectrometry. A prominent peak at *m*/*z* = 149.023 is also observed in the low-energy spectra, which is unequivocally assigned to the protonated phthalic anhydride ion ([C_8_H_5_O_3_]^+^), a ubiquitous diagnostic fragment originating from phthalate plasticizer contamination common in ESI sources.^47^

**Figure 2:**
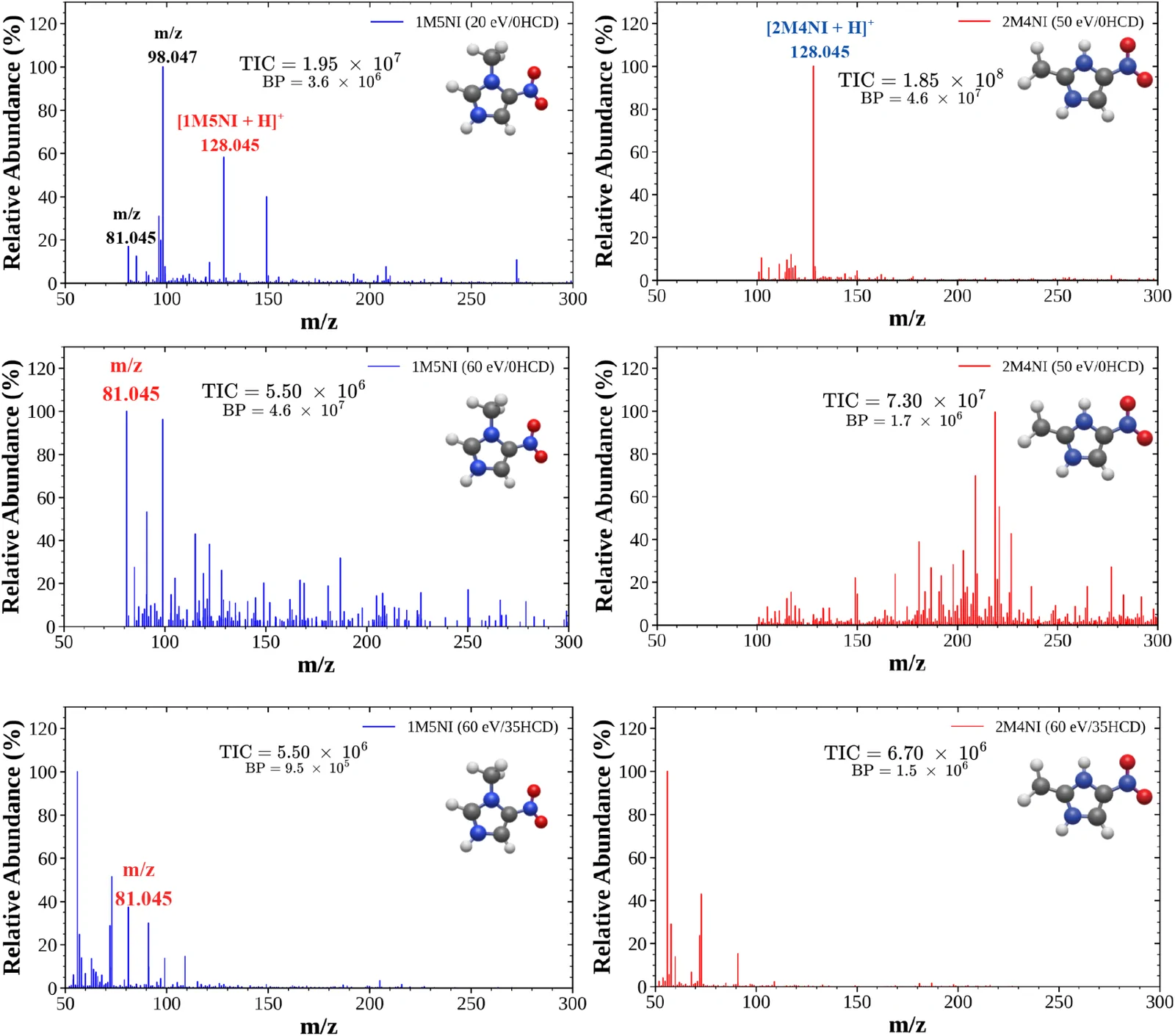
Comparative fragmentation profiles of 1M5NI (left) and 2M4(5)­NI (right). Top: Low-energy regime (20 V) showing the intact protonated precursor ions (*m*/*z* = 128) and fragments (*m*/*z* = 98, 81) in the 1M5NI case. Middle: High-energy spectra optimized for visualization (60 V for 1M5NI and 50 V for 2M4(5)­NI). Bottom: High-energy HCD spectra (60 V, NCE 35) focused on the *m*/*z* 50–150 range. Key diagnostic fragments originating from the analytes are highlighted. **TIC** stands for Total Ion Current, representing the sum of all detected ion intensities. **BP** stands for Base Peak, indicating the absolute intensity of the most abundant ion in the spectrum.

By increasing the IS-CID collision energy to 50 V for 2M4(5)­NI and to 60 V for 1M5NI, the divergence in their fragmentation patterns becomes markedly more pronounced. In this regime, only [1M5NI+H]^+^ exhibits a detectable aromatic structure at *m*/*z* = 81.045, which then predominates in the spectra at higher energies. Notably, under these high-energy conditions, 2M4(5)­NI signals are characterized by nonspecific fragmentation and/or aggregation. The [2M4(5)­NI+H]^+^ spectrum exhibits intense peaks in a high-mass region, indicating polymerization or molecular aggregation, likely arising from ubiquitous ESI­(+) contaminants or uncontrolled clustering events inherent to the high instability of the ring-opened species.

To refine the analysis and focus on the diagnostic mass region—specifically, the window associated with the ejection of cytotoxic radicals such as NO^•^ and OH^•^–both samples were subjected to higher-energy collisional dissociation (HCD, NCE 35) combined with a 60 V IS-CID activation, targeting the *m*/*z* = 50–150 range. The spectra reveal that in this region, typically populated by ubiquitous metal adducts or solvent clusters^48^ only the *m*/*z* = 81.045 peak persists as a product of [1M5NI+H]^+^ in the spectrum. There are no traces of specific [2M4(5)­NI+H]^+^ fragments in this window. This mass range was strictly selected because the Q Exactive Plus Orbitrap operates with a lower mass detection limit of *m*/*z* = 50.

These results indicate that the [2M4(5)­NI+H]^+^ species tends to fragment into small neutral species or ions below the lower mass detection limit (*m*/*z* = 50)^23^ whereas only [1M5NI+H]^+^ possesses an aromatic structure capable of maintaining structural stability under high-energy conditions. This hypothesis is supported by electron ionization (EI) mass spectra available in the SpectraBase database (Figure 3).^14,15^ In these gas-phase EI spectra, the 2M4(5)­NI isomer shows a higher fragmentation tendency than 1M5NI. In particular, unlike in ESI­(+), 2M4(5)­NI in EI presents a high abundance of the species with *m*/*z* = 81. However, we propose that this pattern arises from a distinct mechanistic pathway. The *m*/*z* = 81 fragment in EI probably originates from the direct loss of a neutral nitro radical $\left({\mathrm{NO}}_{2}^{•}\right)$ , whereas in ESI­(+)—specifically for [1M5NI+H]^+^—it stems from the sequential loss of NO^•^ followed by OH^•^.

**Figure 3:**
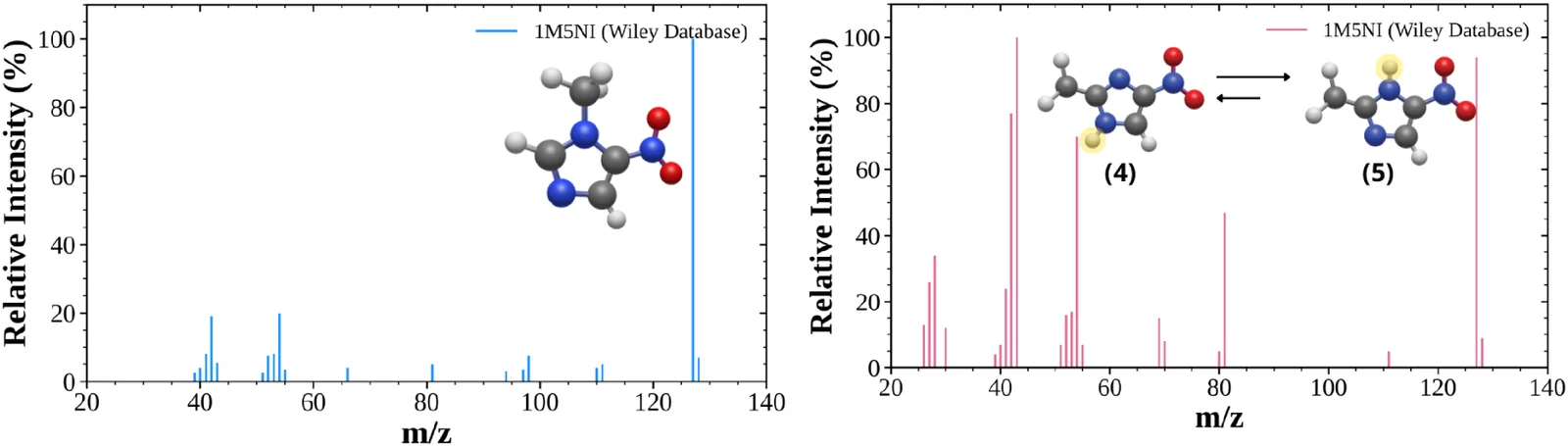
EI mass spectra obtained from the Wiley Mass Spectra Database for 1M5NI (left) and 2M4(5)­NI (right) in the gas phase at 70 eV.

Although the radical molecular ion (M^•+^) with *m*/*z* = 127 has the most intense peak in the EI spectra, both isomers exhibit a signal with *m*/*z* = 128. This indicates a tendency for self-protonation at the unsubstituted nitrogen in the gas phase, a well-documented ion–molecule reaction in EI sources for nitrogen-containing compounds with high proton affinities.^49^ However, beyond *m*/*z* = 128, 1M5NI uniquely displays the *m*/*z* = 98 fragment, in addition to *m*/*z* = 81. Comparing the relative intensities of the *m*/*z* = 81 peak reveals a sharp discrepancy: while it constitutes ≃50% of the base peak intensity for 2M4(5)­NI, it remains below 10% for 1M5NI. Furthermore, in the 1M5NI spectrum, the intensity of the *m*/*z* = 98 peak is slightly higher than *m*/*z* = 81 and comparable to that of the protonated precursor with *m*/*z* = 128. This implies that for 1M5NI, the peaks at *m*/*z* = 98 and 81 originate directly from the protonated fraction ([M + H]^+^) rather than from the radical molecular ion (M^•+^). This argument is reinforced by the dissociation mass balance: for the radical ion with *m*/*z* = 127, there is no direct route to *m*/*z* = 98 via neutral NO^•^ loss, which would yield *m*/*z* = 97. In contrast, starting from the protonated species with *m*/*z* = 128, the loss of NO^•^ yields exactly *m*/*z* = 98, effectively confirming the proton-driven rearrangement mechanism.

In contrast, the EI spectrum of 2M4(5)­NI presents *m*/*z* = 43 as its most intense peak. A combinatorial analysis of the 2M4(5)­NI substituents indicates that no additive combination of the pendant groups can account for this mass while maintaining the ring. Consequently, the formation of *m*/*z* = 43 (likely corresponding to an [H_3_C—C≡NH]^+^ or [HC≡N—OH]^+^ fragment) provides definitive evidence for the rupture of the imidazole ring, which corroborates the hypothesis of structural instability for this isomer.

The fragmentation pathway proposed here for the formation of the *m*/*z* = 81.045 ion, involving the sequential loss of neutral radicals (NO^•^ and OH^•^), is physically consistent with the biologically activated [M + H]^+^ state generated in acidic tumor microenvironments.^50^ Mechanistically, the initial step of this dissociation is triggered by a specific conformational distortion. While the calculated infrared spectra (Figure S5 of the Supporting Information) reveal that both 1M5NI and 2M4(5)­NI exhibit low-frequency in-plane bending modes of the C–N–O angle (δ_C–N–O_), only in 1M5NI does this bending motion effectively project the nitro oxygen toward the adjacent methyl carbon, initiating the isomerization process.

The upper panel of Figure 4 details the multidimensional reaction pathway for the [1M5NI+H]^+^ isomer, elucidating the formation of fragments with *m*/*z* = 98.047 and *m*/*z* = 81.045. Our proposed pathway identifies *m*/*z* = 98.047 as an intermediate of *m*/*z* = 81.045.

**Figure 4:**
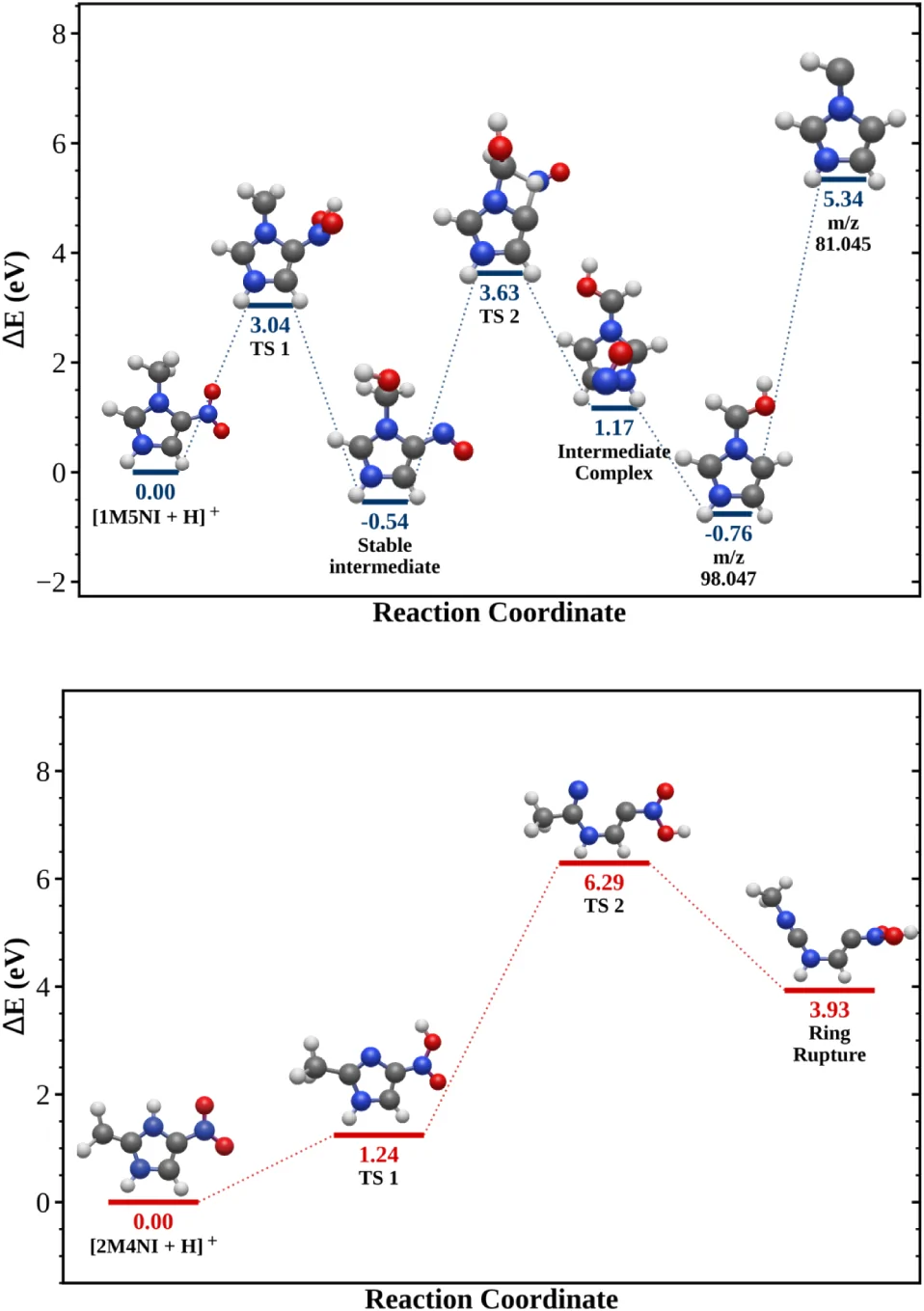
Potential energy surface (PES) scans tracing the reaction coordinates. **Upper panel:** Reaction pathway (Nudged Elastic Band with Transition State Search—NEB-TS—method, CCSD­(T)/cc-pVTZ // ωB97X-D4/def2-TZVP) for 1M5NI detailing the concerted hydrogen transfer, the formation of a stable distonic intermediate, and the subsequent formation of NO^•^ and OH^•^ radicals to yield the *m*/*z* = 98 and *m*/*z* = 81 fragments. **Lower panel:** Analogous coordinate for 2M4(5)­NI (Relaxed Scan Surface, ωB97X-D4/def2-TZVP) demonstrating a frustrated vicinal transfer that channels the internal energy into a catastrophic cleavage of the imidazole ring.

The analysis of the 1M5NI potential energy surface reveals that the initial rearrangement is a concerted process. The transfer of a hydrogen atom from the methyl group to the proximal oxygen atom of the nitro group to form an -NO_2_H group is facilitated not only by the δ_C–N–O_ bending but also necessitates the structural rotation of the adjacent −CH_3_ group to properly align the interacting atoms. Subsequent migration of the hydroxyl group to the electron-deficient methyl carbon center consolidates the formation of a distonic intermediate species, which is more stable than the original protonated isomer by approximately 0.54 eV. The dissociation of the neutral nitric oxide radical (NO^•^) from this complex leads to the fragment ion with *m*/*z* = 98.047. High-level CCSD­(T) calculations reveal that this exergonic pathway has a total activation energy of 3.8 eV. From this preorganized *m*/*z* = 98.047 structure, a subsequent fragmentation occurs via the loss of a hydroxyl radical (OH^•^) to generate the species with *m*/*z* = 81.045, a step that faces a significantly higher energy barrier of 5.4 eV.

It must be emphasized that while activation barriers of 3.8 and 5.4 eV are strictly prohibitive for ground-state thermal biochemical processes, they are accessible during the targeted application of radiotherapy. The massive, localized energy deposition from ionizing radiation provides the energy necessary for this reaction to occur, even *in vivo*. Experimentally, these radiotherapeutic conditions and energetic thresholds are mimicked in our in-source collision-induced dissociation (IS-CID) conditions. As demonstrated in the Supporting Information, for the [1M5NI+H]^+^ precursor (*m*/*z* = 128), an E_*lab*_ of 20 eV yields a single-collision E_*COM*_ of approximately 3.6 eV. This available energy is remarkably close to the 3.8 eV threshold required for the *m*/*z* = 98 pathway, explaining its predominance at lower collision energies under a multiple-collision regime. Conversely, the 5.4 eV barrier for the *m*/*z* = 81 species is energetically disfavored under these mild conditions; it becomes accessible only to a small fraction of the ion population, which accurately accounts for its minor relative abundance (∼10%) in the 20 V spectrum. However, increasing the activation energy to 60 V (*E*_COM_ ≈ 10.7 eV) provides sufficient internal energy for the ion population to overcome the barrier associated with the release of the neutral OH^•^ radical. This drives the sequential structural rearrangements, culminating in the formation of the *m*/*z* = 81.045 fragment, which becomes the predominant peak originating from 1M5NI.

We note that a previous ESI-Q-TOF study by Pandeti et al. investigated the low-energy collision-induced dissociation, using Argon as the collision gas at a pressure of 5 × 10^–5^ mbar, of protonated 1M5NI, assigning the formation of the *m*/*z* = 81 fragment to a direct, concerted loss of a neutral HONO molecule.^51^ Furthermore, their study reported a fragment at *m*/*z* = 82 corresponding to direct ${\mathrm{NO}}_{2}^{•}$ loss, while entirely failing to detect the *m*/*z* 98 intermediate for 1M5NI. Utilizing the sub-5 ppm mass accuracy of the Orbitrap analyzer across a broad range of collision energies (20 to 60 eV) combined with efficient thermal activation in the transfer capillary (320 °C), our data offers a mechanistic route for more energetic profiles, closely mimicking the kinetic energies of secondary electrons generated during clinical radiotherapy.^52^ We unambiguously isolated the *m*/*z* = 98.047 fragment, which was absent in the previous low-energy spectra. Our exact mass balance, coupled with the rigorous CCSD­(T) energetic profile (Figure 4), strongly suggests that the *m*/*z* = 81 species does not arise from a single-step HONO elimination, which would require a structurally distinct and higher-energy transition state. Instead, it is the product of a sequential, energy-dependent dissociation pathway: the initial rearrangement and release of an NO^•^ radical to form the stable intermediate at *m*/*z* = 98, followed by the high-energy ejection of an OH^•^ radical. The absence of the *m*/*z* = 82 peak in our high-energy HCD window further shows that, upon energetic activation, the proton-driven rearrangement strictly channels the molecular dissociation toward the sequential release of these specific cytotoxic radicals.

Finally, analysis of the ADCH charge population^53^ in the dissociation complex confirms the nature of the fragmentation: the partial charge in the leaving nitroso group is approximately +0.08 au (Figure S3), consistent with the elimination of a neutral radical and the retention of positive charge in the imidazole ring (forming the 1-formyl-imidazole cation, *m*/*z* = 98). Stabilization of this product by about 1.0 eV relative to the 1M5NI precursor reinforces the viability of this pathway.

The lower panel of Figure 4 reveals the impossibility of [2M4(5)­NI+H]^+^ following a mechanism analogous to that of 1M5NI. The reaction mechanism scan culminates with the cleavage of the imidazole ring.

Structurally, the fifth geometry of the 2M4(5)­NI scan resembles the first transition state (TS1) of 1M5NI. This step is characterized by the attempted approach of the hydroxyl group (from the -NO_2_H moiety) toward the vicinal center. In 1M5NI, this center is the reactive methylene carbon (−CH_2_), whereas in 2M4(5)­NI, it is the adjacent ring nitrogen. While in 1M5NI this migration is successfully enabled by the *ortho* effect, in 2M4(5)­NI it represents a frustrated vicinal transfer.^6,7^

The physical basis for this hindered pathway lies in the molecular electrostatic potential (MESP) topography (Figure S4). The evaluation of the electrostatic surface for 2M4(5)­NI reveals a highly repulsive region for the hydroxyl attack localized at unsubstituted nitrogen. In contrast, this repulsive potential is significantly mitigated around the reactive methyl carbon in 1M5NI. This difference arises because the unhindered unsubstituted nitrogen in 2M4(5)­NI fully exposes its lone electron pair directly toward the migration path, electrostatically blocking the hydroxyl attack.

This electrostatic behavior demonstrates that reactivity analysis based on the 3D MESP is fundamentally superior to the accounting of atomic partial charges.^54^ An analysis focused solely on ADCH charges^53^ could be misleading: the carbon atom of the CH_2_ group in 1M5NI exhibits a much more negative atomic charge (−0.46 au) (Atom 1 in Figure S1) than the free nitrogen in 2M4(5)­NI (−0.27 au) (Atom 2 in Figure S2), which would incorrectly suggest a greater intrinsic repulsion in 1M5NI. However, the MESP describes the reactivity more reliably by mapping the three-dimensional spatial distribution of the electrostatic field via Coulomb’s law. The spatial projection reveals that the partial negative charge on the CH_2_ carbon in 1M5NI is shielded by its peripheral hydrogen atoms, whereas the negative charge density on the nitrogen in 2M4(5)­NI is fully exposed. A comparison of these electrostatic potential iso-surfaces is provided in Figure S4 of the Supporting Information.

Upon comparison of the mass spectrum from this work (Figure 2) with the Wiley database for the 2-methyl-4(5)-nitroimidazole isomer, the absence of the *m*/*z* = 81 peak in our spectrum is notable. Under our experimental conditions, the molecule is protonated ([M + H]^+^), which ensures the presence of hydrogen atoms on both ring nitrogens. Consequently, the molecule follows a reaction pathway analogous to that of the neutral 2M5NI tautomer: the initial distortion brings the nitro group closer to the adjacent nitrogen, allowing the capture of the vicinal hydrogen and the formation of an −NO_2_H group. However, as demonstrated by the topological analysis of the electrostatic potential, the subsequent migration of the hydroxyl group encounters severe electrostatic repulsion, preventing the OH group from attacking nitrogen. This electrostatic hindrance prevents the lower-energy route, meaning that the higher-energy cleavage of the imidazole ring becomes the primary pathway, precluding the formation of the *m*/*z* = 81 fragment.

However, the Wiley spectrum is generated via electron ionization in the gas phase, where the neutral sample exists as an equilibrium mixture of the 2M4NI and 2M5NI tautomers. Based on the experimental data and the energy profiles in the lower panel of Figure 4, we propose that the fragment with *m*/*z* = 81 observed in the database originates exclusively from the 2M4NI tautomer. Due to the lack of an available vicinal hydrogen on the adjacent nitrogen (which has a lone electron pair), the reaction pathway via rearrangement is nonviable. The energy transferred is then allocated to direct cleavage of the C–NO_2_ bond, culminating in the expulsion of the neutral ${\mathrm{NO}}_{2}^{•}$ radical. To compensate for the electron deficiency left on the carbon of the ring, an internal hydrogen transfer occurs from the neighboring carbon, ensuring the survival of the intact imidazole ring (*m*/*z* = 81). This direct cleavage process is energetically more costly than hydrogen abstraction but is the only accessible route for 2M4NI.

We propose that the peak at *m*/*z* = 43, which dominates the Wiley spectrum (but is not observed in our work as it falls below the low-mass cutoff of the Orbitrap analyzer), corresponds to a fragmentation product of the 2M5NI tautomer. The presence of a vicinal hydrogen donor renders the initial transfer highly favorable, directing the 2M5NI molecule into a reaction pathway analogous to that of our protonated model. As demonstrated by our calculations, this pathway inevitably leads to the cleavage of the imidazole ring.

Thus, it is concluded that the initial distortion is activated in both classes of structures (1M5NI and 2M5NI analogs). However, the presence of the methyl group adjacent to NO_2_ in 1M5NI provides the appropriate electrostatic surface not only for hydrogen abstraction but also for the effective migration of the hydroxyl group toward the formation of a stable intermediate. It is due to this electrostatic viability of 1M5NI that this species can maintain ring integrity, enabling the sequential ejection of neutral radicals (NO^•^ and OH^•^), high redox potential species crucial for the efficacy of 1M5NI as a radiosensitizing agent.

To reconcile the classical requirement for a high redox potential with the observed gas-phase stability of these molecules, we must consider the physiological microenvironment. Classical quantitative structure–activity relationship (QSAR) models, despite their inherent simplifications, have historically emphasized that a high one-electron reduction potential is essential for the initial capture of radiolytic electrons by nitroaromatics.^9,10^ However, recent gas-phase studies have demonstrated that isolated radical anions of methylated nitroimidazoles, formed via low-energy electron attachment, actively resist dissociation.^16^ This paradox can be explained by quantum dynamics of the capture process. During clinical radiotherapy, the attenuation of the primary high-energy ionizing beam by the aqueous biological medium generates a massive cascade of low-energy secondary electrons.^52^ While the neutral nitroimidazole molecules readily capture these thermalized electrons because of their high electron affinity, this attachment fails to populate a dissociative resonance state capable of spontaneously cleaving the molecular framework. Consequently, electron capture alone is insufficient to trigger the release of the cytotoxic radicals.

In a biological context, however, hypoxic tumor microenvironments are distinctly acidic.^50^ Driven by the increase in basicity following one-electron reduction^11,19^ any initially formed radical anion undergoes rapid protonation. This protonated state, which directly parallels the [M + H]^+^ species investigated in our ESI­(+) experiments, serves as the structural trigger. As our energetic profiles reveal, the protonated state shifts the energetic landscape, making the multidimensional rearrangement the prevalent and accessible pathway for 1M5NI. This favored pathway channels the internal energy to preserve the imidazole core while efficiently expelling the neutral NO^•^ and OH^•^ radicals. Therefore, we propose that the radiosensitizing efficacy of 1M5NI is not dictated solely by the initial electron affinity of the neutral species but depends intrinsically on the subsequent proton-activated fragmentation pathways that only become accessible within the acidic biological medium.

## Conclusion

We concluded that for a nitroimidazole derivative to be a promising radiosensitizer candidate, it must possess the structural capacity to undergo an ortho- or vicinal rearrangement. We propose that this rearrangement is initially triggered by a conformational distortion involving the nitro group, which subsequently drives a multidimensional reaction pathway. Furthermore, for this process to be initiated, the neutral species must possess sufficient electron affinity to form a highly basic radical anion capable of rapid protonation within a mildly acidic tumor microenvironment.

By correlating our findings with established literature, we conclude that 1M5NI acts as a potent radiosensitizer in hypoxic tumors because it undergoes rapid protonation following the formation of a highly basic radical anion. This sequence—triggered by the high electron affinity of the neutral species and subsequent interaction with the acidic microenvironment—fundamentally shifts the energetic landscape toward proton-activated dissociation. Crucially, the substantial energy deposited by clinical radiotherapy provides the necessary driving force to traverse these high-activation barriers *in vivo*. Consequently, the molecule effectively ejects cytotoxic NO^•^ and OH^•^ radicals, surpassing the inherent dissociation resistance of the isolated anionic species while maintaining a stable imidazole scaffold. This proposed mechanism not only elucidates the efficacy of 1M5NI but also establishes essential criteria for designing superior radiosensitizers: candidates must possess high electron affinity to promote initial reduction, a structural configuration capable of undergoing an ortho-mediated multidimensional rearrangement, and the capacity to leverage radiotherapeutic energy to unlock their cytotoxic potential.

## Supplementary Material



## Notes

Kinematic calculations for HCD fragmentation and center-of-mass energy; calculated infrared (IR) spectra highlighting the relevant bending modes (Figure S5); Cartesian coordinates, unscaled harmonic vibrational frequencies, zero-point energies (ZPE), and absolute energies for all optimized stationary points (PDF)
