# Supplementary material for: Dissociation Dynamics of Protonated Nitroimidazoles: The Ortho Effect as the Mechanistic Trigger for Radical Formation
Source: J Phys Chem A. 2026 Oct 22;130(44):8763–71. doi: 10.1021/acs.jpca.6c04617 (PMC13746804; doi:10.1021/acs.jpca.6c04617)
Supplement: Supplementary file 1 [file jp6c04617_si_001.pdf]

Supporting Information: Dissociation Dynamics of Protonated Nitroimidazoles: The Ortho Effect as the Mechanistic Trigger for Radical Formation

Jorge H. C. Basilio,[†] Ricardo R. Oliveira,[*,‡] Fábio C. S. Nogueira,[‡] Nina D. Ortiz,[‡] Gilberto B. Domont,[‡] Alexander Dorn,[¶] and Wania Wolff[*,†]

†*Physics Institute, Federal University of Rio de Janeiro, Rio de Janeiro, RJ 21941-909, Brazil.*

‡*Chemistry Institute, Federal University of Rio de Janeiro, Rio de Janeiro, RJ 21941-909, Brazil.*

¶*Max Planck Institute for Nuclear Physics, Saupfercheckweg 1, 69117, Heidelberg, Germany.*

E-mail: rrodrigues.iq@gmail.com; wania@if.ufrj.br

Contents

1 Vibrational Frequencies and Fermi Resonance Correction S-3

2 Partial Charges (ADCH) S-6

3 Molecular Electrostatic Potential (MESP) Surfaces S-10

4 Relative Stabilities of the Neutral Isomers S-11

5 Relative Stabilities of the protonated isomers S-11

6 Data: Cartesian Coordinates and Absolute Energies for Upper Panel of Figure 4 S-12

7 Data: Cartesian Coordinates and Absolute Energies for Lower Panel of Figure 4 S-17

8 Kinematic Considerations for HCD Fragmentation S-20

9 Infra-red spectra for $[1M5NI+H]^{+}$ and $[2M4NI+H]^{+}$ S-21

References S-23

1 Vibrational Frequencies and Fermi Resonance Correction

As discussed in the main text, an intrinsic scaling factor ($\lambda = 0.988$) was derived to correct for the accidental Fermi resonance observed in the 1M5NI isomer at the ωB97X-D4/def2-TZVP level of theory. Table S1 details the harmonic and anharmonic frequencies.

Table S1: Comparison between Harmonic (ω) and Anharmonic (ν) frequencies for [1M5NI + H]$^+$. Values in red indicate Fermi Resonances.

Mode	ω_{harm} (cm^{-1})	ν_{anharm} (cm^{-1})	Ratio (ν/ω)
0	51.93	43.69	0.8414
1	182.95	167.38	0.9149
2	207.54	181.36	0.8739
3	228.51	224.66	0.9831
4	256.72	247.50	0.9641
5	349.48	343.54	0.9830
6	433.62	394.77	0.9104
7	550.27	543.07	0.9869
8	621.21	616.58	0.9925
9	640.46	623.09	0.9729
10	691.44	670.72	0.9700
11	716.47	1026.10	1.4321
12	772.15	766.21	0.9923
13	865.93	829.12	0.9575
14	888.49	874.43	0.9842
15	918.13	899.06	0.9792
16	946.92	936.20	0.9887
17	1100.78	1078.71	0.9799
18	1120.96	1100.06	0.9814
19	1158.76	1141.86	0.9854
20	1158.99	1140.95	0.9844
21	1228.93	1206.19	0.9815
22	1283.90	1258.74	0.9804
23	1352.16	1308.84	0.9680
24	1433.13	1725.64	1.2041
25	1453.00	1424.95	0.9807
26	1472.34	1441.64	0.9791

Continued on next page

Table S1 – Continued from previous page

**Mode**	ω_{harm} (cm^{-1})	ν_{anharm} (cm^{-1})	**Ratio** (ν/ω)
27	1481.11	1437.85	0.9708
28	1509.40	1472.00	0.9752
29	1521.78	1485.61	0.9762
30	1622.83	1581.72	0.9747
31	1668.52	1638.15	0.9818
32	1726.23	1694.42	0.9816
33	3113.74	3019.65	0.9698
34	3212.61	3080.21	0.9588
35	3219.98	3084.43	0.9579
36	3296.54	3197.71	0.9700
37	3312.42	3185.82	0.9618
38	3629.95	3478.69	0.9583
**Average Intrinsic Scaling Factors**			
**Low Freq.** (< 2000 cm^{-1})	-	-	**0.9883** (±0.0943)
**High Freq.** (> 2000 cm^{-1})	-	-	**0.9628** (±0.0052)

Table S2: Comparison between Harmonic (ω) and Anharmonic (ν) frequencies for [2M4(5)NI + H]$^+$. data.

**Mode**	ω_{harm} (cm^{-1})	ν_{anharm} (cm^{-1})	**Ratio** (ν/ω)
0	38.24	-7.34	-0.1918
1	86.34	92.20	1.0679
2	159.10	157.50	0.9899
3	175.50	174.85	0.9963
4	296.14	297.94	1.0061
5	346.36	348.24	1.0054
6	422.73	420.49	0.9947
7	551.51	544.84	0.9879
8	610.93	603.26	0.9874
9	681.27	675.15	0.9910
10	696.63	687.72	0.9872
11	698.22	692.20	0.9914
12	756.27	745.08	0.9852
13	774.43	768.10	0.9918
14	852.04	845.95	0.9929
15	895.61	882.18	0.9850
16	1005.50	988.60	0.9832
17	1036.78	1014.22	0.9782

Continued on next page

Table S2 – Continued from previous page

**Mode**	ω_{harm} **(cm^{-1})**	ν_{anharm} **(cm^{-1})**	**Ratio** (ν/ω)
18	1048.20	1033.35	0.9858
19	1071.29	1048.98	0.9792
20	1130.13	1110.98	0.9831
21	1204.69	1179.53	0.9791
22	1301.02	1270.40	0.9765
23	1345.30	1310.17	0.9739
24	1436.09	1406.73	0.9796
25	1446.09	1411.78	0.9763
26	1458.39	1429.59	0.9803
27	1480.21	1448.88	0.9788
28	1483.05	1440.61	0.9714
29	1554.16	1537.43	0.9892
30	1628.89	1599.34	0.9819
31	1706.96	1671.79	0.9794
32	1726.10	1693.18	0.9809
33	3082.46	2982.03	0.9674
34	3159.71	3027.01	0.9580
35	3184.50	3050.63	0.9580
36	3312.21	3179.49	0.9599
37	3598.50	3440.65	0.9561
38	3631.22	3479.97	0.9583
**Average Intrinsic Scaling Factors**			
**Low Freq.** (< 2000 cm^{-1})	-	-	**0.9523** ($\pm$0.2029)
**High Freq.** (> 2000 cm^{-1})	-	-	**0.9596** ($\pm$0.0037)

2 Partial Charges (ADCH)

This section lists the partial charges calculated using the At omic Dipole Moment Corrected Hirshfeld (ADCH) method. The molecular figures show the numbering of each atom presented in the partial charge calculation table.

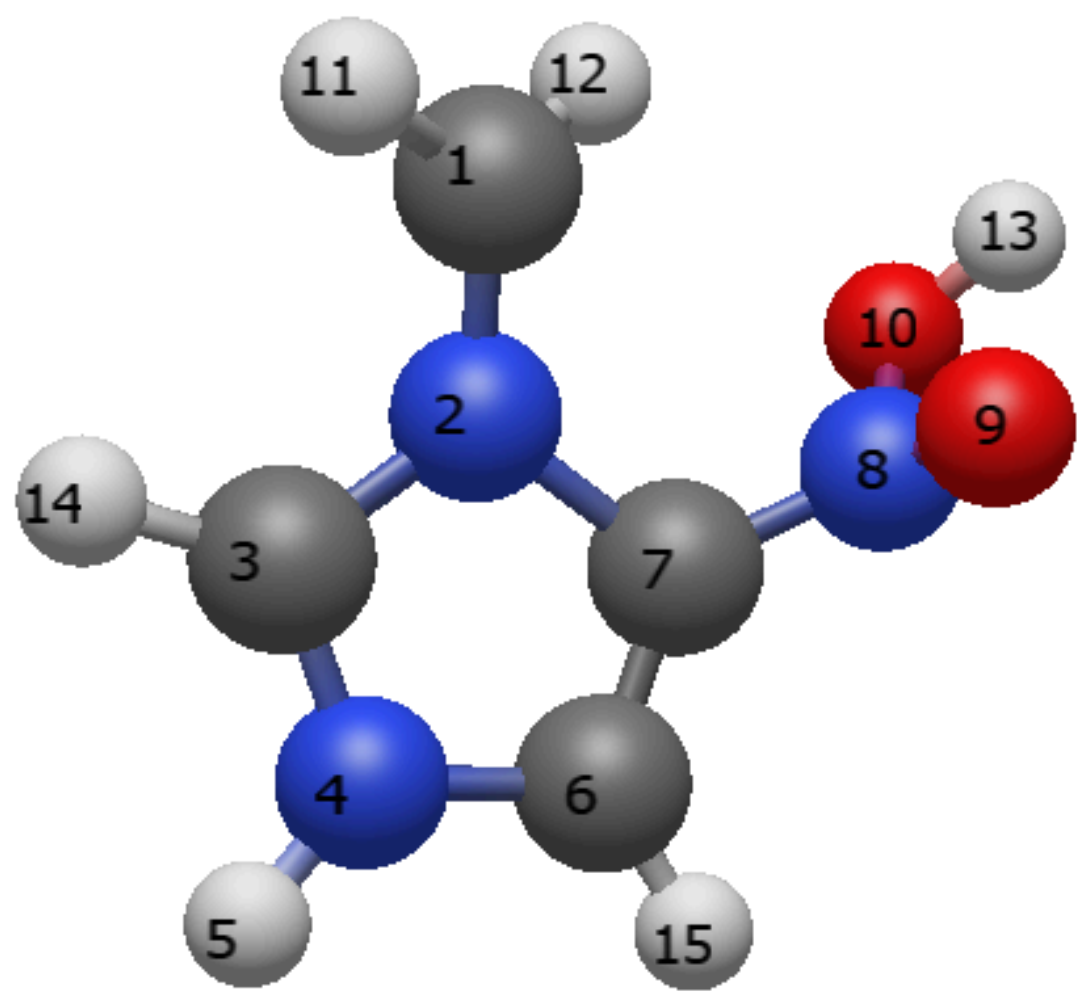

Figure S1: First Transition State (TS1) for 1M5NI reaction. Labels on the atoms are the same as those in the partial charge tables

```
*Atom    1(C ):    -0.46290484*
Atom    2(N ):     0.10588570
Atom    3(C ):     0.05528841
Atom    4(N ):    -0.10205692
Atom    5(H ):     0.33911509
Atom    6(C ):     0.04256428
Atom    7(C ):    -0.06971601
Atom    8(N ):     0.33390699
Atom    9(O ):    -0.23210507
Atom   10(O ):    -0.30185418
Atom   11(H ):     0.25437207
Atom   12(H ):     0.25773680
Atom   13(H ):     0.41986281
Atom   14(H ):     0.18303115
Atom   15(H ):     0.17687371
```

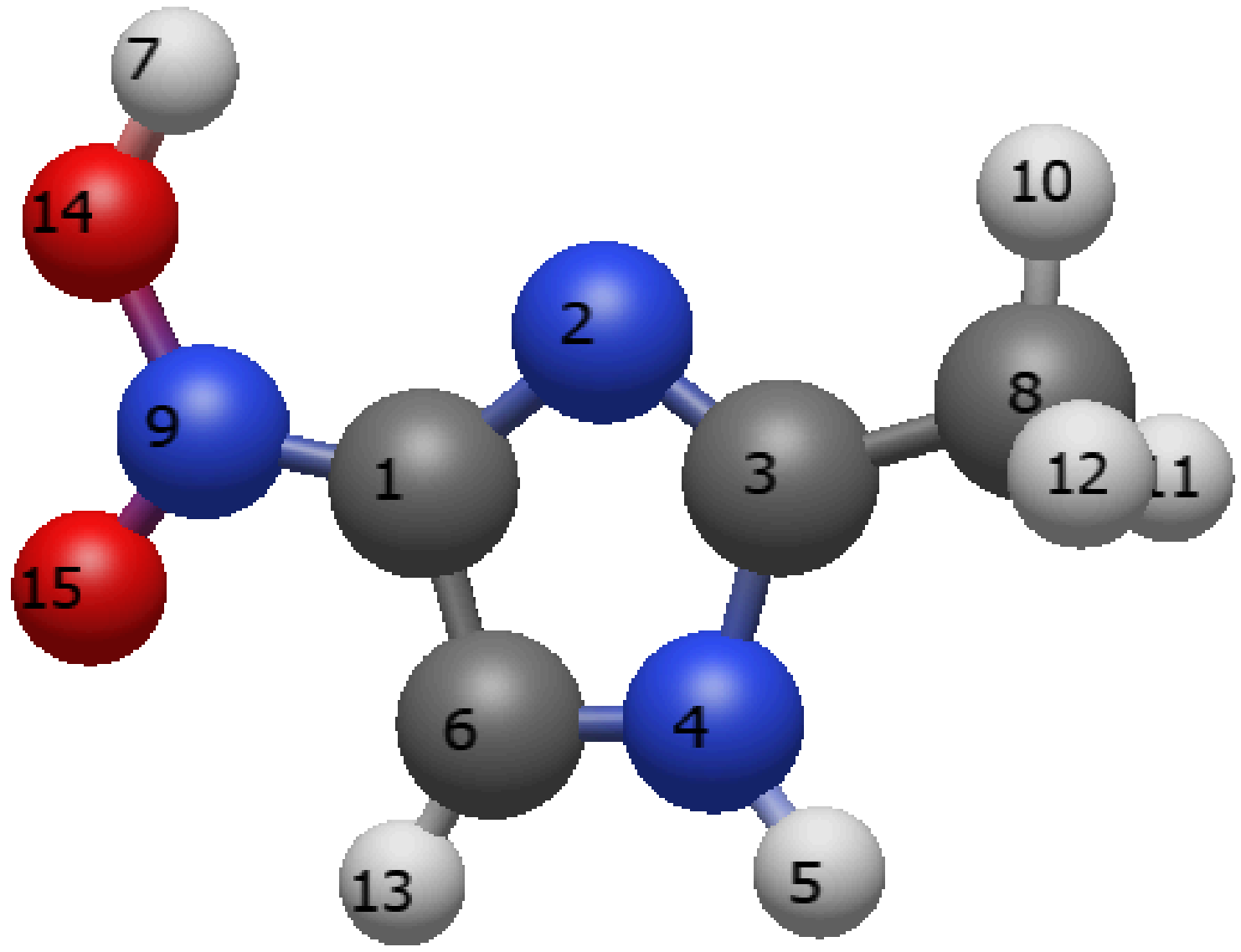

Figure S2: analogous to TS1 from 1M5NI to 2M4NI. Labels on the atoms are the same as those in the partial charge tables

Atom	1(C):	0.03907550
* Atom	2(N):	-0.27535033*
Atom	3(C):	0.13900156
Atom	4(N):	-0.05507048
Atom	5(H):	0.31917226
Atom	6(C):	-0.04097290
Atom	7(H):	0.41514451
Atom	8(C):	-0.25586823
Atom	9(N):	0.37522426
Atom	10(H):	0.15613487
Atom	11(H):	0.14066316
Atom	12(H):	0.14064276
Atom	13(H):	0.21392909
Atom	14(O):	-0.21417507
Atom	15(O):	-0.09755096

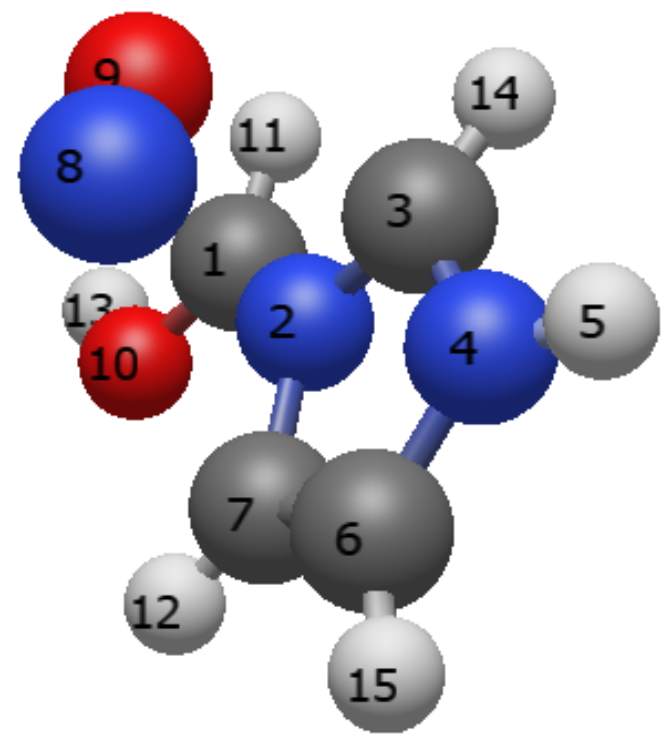

Figure S3: Stable Complex for the 1M5NI reaction for the NO elimination. Labels on the atoms are the same as those in the partial charge tables

Atom	1(C):	0.04624808
Atom	2(N):	0.23709181
Atom	3(C):	-0.08034979
Atom	4(N):	-0.71510708
Atom	5(H):	0.63601553
Atom	6(C):	0.09142468
Atom	7(C):	-0.12724028
*Atom	8(N):	0.01348311*
*Atom	9(O):	0.06873927*
Atom	10(O):	-0.35979766
Atom	11(H):	0.20072088
Atom	12(H):	0.16876225
Atom	13(H):	0.45129277
Atom	14(H):	0.19036100
Atom	15(H):	0.17835542

3 Molecular Electrostatic Potential (MESP) Surfaces

The topological analysis of the electrostatic potential reveals the severe repulsive barrier in 2M4NI compared to the sterically shielded carbon center in 1M5NI.

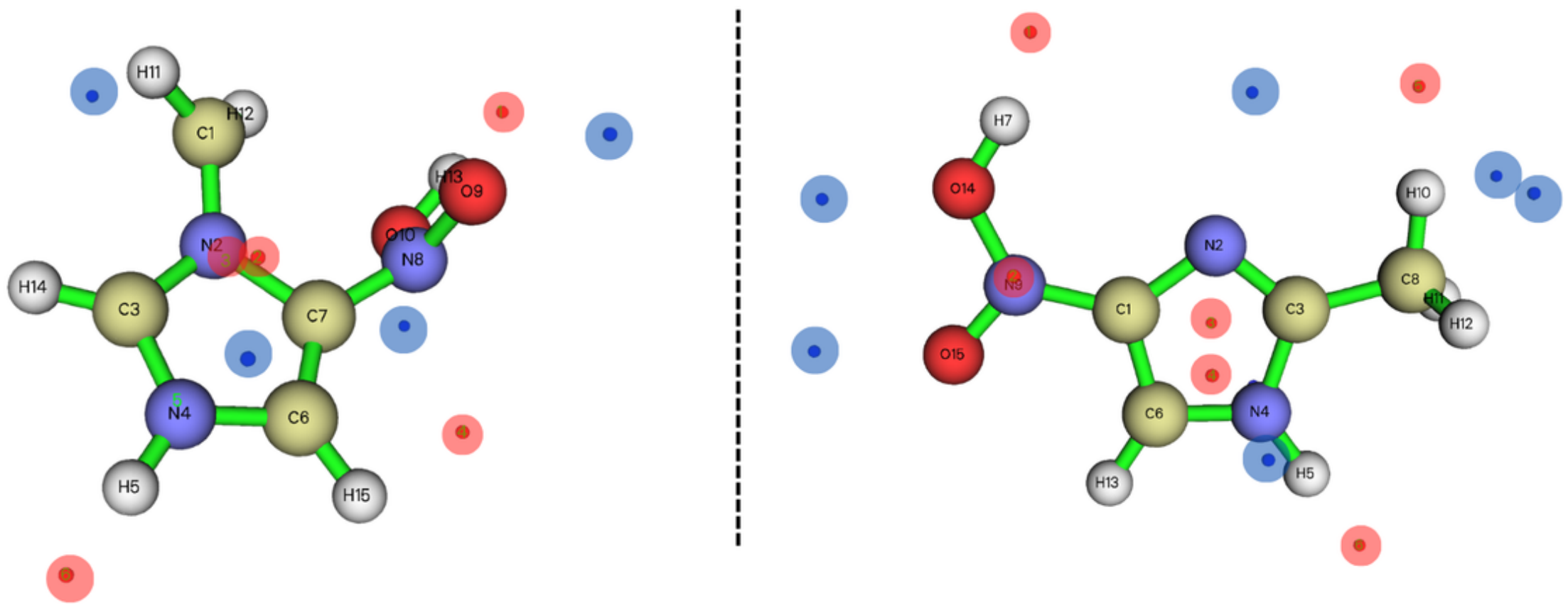

Figure S4: Molecular Electrostatic Potential (MESP) mapped onto the 0.001 a.u. electron density isosurface for the transition state geometry of the $[1M5NI{+}H]^+$ (left) and the $[2M4(5)NI{+}H]^+$ (right) analogue structure. Red regions/spheres indicate local maxima (highest positive potential, strongly repulsive), whereas blue regions/spheres represent local minima (lowest positive potential, electronically accessible). The methyl carbon vicinity in 1M5NI presents a clear minimum, favoring the attack, whereas the corresponding nitrogen region in 2M4NI is dominated by a repulsive barrier inside the imidazolic ring.

4 Relative Stabilities of the Neutral Isomers

To evaluate the inherent structural instability driven by steric hindrance without the influence of the protonated ring, geometry optimizations of the neutral 1M5NI and 2M4NI species were performed at the DFT level (ωB97X-D4/def2-TZVP).

Table S3: Absolute energies and relative stabilities of the neutral isomers.

**Isomer**	**Zero-Point Corrected Energy (Hartree)**	**ΔE (kcal/mol)**
2M4NI	-470.3683	0.00
2M5NI	-470.3679	0.27
1M5NI	-470.3521	10.15

5 Relative Stabilities of the protonated isomers

Table S4: Absolute energies and relative stabilities of the protonated isomers obtained with CCSD(T).

**Isomer**	**Zero-Point Corrected Energy (Hartree)**	**ΔE (kcal/mol)**
$[2M5NI + H]^+$	-469.66609	0.00
$[1M5NI + H]^+$	-469.65007	10.05

6 Data: Cartesian Coordinates and Absolute Energies for Upper Panel of Figure 4

The following tables list the optimized Cartesian coordinates (in Å) and the Zero-Point Energy for all highlighted points in the upper panel of figure 4. The structures were optimized at the ωB97X-D4/def2-TZVP level of theory. The energies were obtained with CCSD(T)

[1M5NI+H]$^+$ (Precursor Ion)

$E_{ZPE} = -469.6500$ Hartree

C	2.31671955730945	-4.44853852773057	-0.98888770493948
N	3.30971237078320	-3.58769968974562	-0.31104080383911
C	4.36022045887807	-4.04939302465499	0.34879543746913
N	5.05355183832150	-3.01992210728217	0.84024318862916
H	5.90131619966038	-3.10358588234187	1.38495004019994
C	4.44042773467937	-1.84532038540030	0.49322146020255
C	3.35186597538689	-2.20746464695208	-0.22598102186856
N	2.37409443782741	-1.31000234542627	-0.82048928429133
O	2.57902507810304	-0.13712403321875	-0.63913638439953
O	1.46312311113166	-1.82018545743559	-1.43060753126411
H	2.62504404299335	-5.48069457496703	-0.83331720855779
H	2.29973263279818	-4.21061052732262	-2.04923004845553
H	1.33521042432869	-4.27363446442489	-0.55613987133996
H	4.61268276191548	-5.09231453783956	0.46822896937759
H	4.80257337588331	-0.86782979525767	0.76838076307703

TS1 (1M5NI ortho-effect rearrangement)

$E_{ZPE} = -469.5381$ Hartree

Imaginary frequency = -214.48 cm^{-1}

C	-0.95137353563513	-1.50928865396486	-0.37774934655294
N	0.10921968664037	-0.72575352625628	-0.07038787340608
C	1.21567318852225	-1.06877801186639	0.60085163303781
N	1.83033832863304	0.03535313015321	1.04856935980672
H	2.72730778840468	0.02983301629405	1.51352397923796
C	1.16339438172429	1.15776411655333	0.65763834116218
C	0.10575462535018	0.73400628082717	-0.07211953829001
N	-1.07081921229141	1.30756395092957	-0.46012470767717
O	-2.02123484643715	1.37439347013617	0.30839022686656
O	-1.32609837543202	1.09555203064125	-1.78078132417983
H	-1.13707436923229	-2.36744436257323	0.24883247065149
H	-1.44237184219661	-1.36246654443906	-1.32503711038787
H	-2.29144411139110	1.21613135100226	-1.86631854178041
H	1.59506053869382	-2.07395109318969	0.68452400541046
H	1.49366775464709	2.15708484575249	0.89018842610113

Stable intermediate

$E_{ZPE} = -469.6697$ Hartree

C	1.87915606532092	-2.64214710046869	-1.92133087035137
N	2.90254430739870	-2.35970339893353	-0.86131378153676
C	3.34206927727341	-3.19901736445514	0.05948308047932

N	4.18494281514351	-2.54213076529948	0.86213153874830
H	4.67153524321523	-2.95476185980628	1.64645559043897
C	4.29018200750068	-1.23646685571450	0.45382215462628
C	3.47823879205835	-1.12329771556208	-0.63018580817993
N	3.19267689374837	-0.03468555218561	-1.53193573912144
O	3.70035797774148	0.98985040665969	-1.19033237155390
O	0.68554626321807	-2.03599942940864	-1.60655889787667
H	1.82805262819909	-3.72992424050350	-2.01174766837055
H	2.26537160715922	-2.20357613174596	-2.83978444315625
H	0.10109764920831	-2.63257516273169	-1.12672904937439
H	3.07232167201637	-4.24073197648679	0.15083279092675
H	4.92021380079808	-0.50851885335765	0.93956347430168

TS2 (NO fragmentation)

$E_{ZPE} = -469.5165$ Hartree

Imaginary frequency = -1849.05 cm^{-1}

C	-0.951000	-0.288323	-1.076056
N	-0.056761	-0.391084	0.066553
C	0.488533	-1.289126	0.876838
N	1.182772	-0.593971	1.776993
H	1.709391	-1.013693	2.530582
C	1.071615	0.771475	1.531493
C	0.269067	0.903793	0.438924
N	0.444981	1.406119	-1.909110
O	0.187943	2.352325	-2.421261
O	-2.240985	-0.495693	-0.697361

H	-0.631852	-0.850397	-1.954798
H	-0.637231	1.086217	-0.709332
H	-2.788373	-0.729195	-1.456648
H	0.383848	-2.362331	0.845502
H	1.568053	1.493883	2.157681

Complex intermediate

$E_{ZPE} = -469.6069$ Hartree

C	1.776769	-3.133957	-1.493957
N	2.650950	-2.529945	-0.744995
C	4.029207	-2.376226	-1.021024
N	4.558833	-1.728799	0.045253
H	5.542288	-1.608061	0.217708
C	3.545732	-1.296278	0.875837
C	2.369764	-1.763044	0.427768
N	4.071400	0.280949	-1.449531
O	3.746061	-0.581948	-2.110308
O	0.506820	-3.052040	-1.146622
H	2.097722	-3.693916	-2.364779
H	1.366749	-1.656897	0.799708
H	-0.064178	-3.551099	-1.744979
H	4.569674	-3.072431	-1.642968
H	3.746514	-0.689993	1.745256

[1M5NI+H - NO] (m/z 98.047)$^+$

$E_{ZPE} = -469.6780$ Hartree

C	1.773259	-3.151887	-1.546007
N	2.692012	-2.524657	-0.722799
C	4.023698	-2.531804	-0.878112
N	4.551378	-1.779122	0.090878
H	5.539410	-1.612577	0.210739
C	3.548256	-1.280901	0.887419
C	2.377728	-1.740837	0.387754
O	0.519848	-3.096777	-1.094037
H	2.114694	-3.844539	-2.301595
H	1.359251	-1.594686	0.702367
H	-0.090427	-3.533671	-1.700795
H	4.566764	-3.050751	-1.651286
H	3.745544	-0.647048	1.736321

[1M5NI+H - NO - OH]$^+$ (m/z 81.045)

$E_{ZPE} = -469.4537$ Hartree

C	1.930501	-3.675037	-1.226516
N	2.785040	-2.807323	-0.597302
C	4.080901	-2.505991	-0.906718
N	4.511091	-1.630708	-0.039486
H	5.443696	-1.234081	-0.037115
C	3.499743	-1.321343	0.883671

C	2.430170	-2.052110	0.535688
H	2.506010	-4.102667	-2.067249
H	1.439127	-2.138757	0.951832
H	4.636689	-2.929481	-1.731778
H	3.659529	-0.615680	1.682686

7 Data: Cartesian Coordinates and Absolute Energies for Lower Panel of Figure 4

The following tables list the optimized Cartesian coordinates (in Å) and the Zero-Point Energy for all highlighted points in the lower panel of figure 4. The structures were optimized at the ωB97X-D4/def2-TZVP level of theory.

[2M4(5)NI+H]$^+$

$E_{ZPE} = -470.7239$ Hartree

C	2.35550072230609	0.05712477226003	-0.09107252749417
N	3.21176337484992	-1.02005295699393	-0.09700982398038
C	4.46706810501342	-0.58194591602518	-0.09236811116124
N	4.40212104414074	0.75655782949697	-0.08643137996732
H	5.21090516183876	1.36308082358866	-0.08352625321908
C	3.09083162033040	1.18633505972116	-0.08476676727447
H	2.90473237959654	-1.97172899970446	-0.10369365031467
C	5.69903945442716	-1.40821259101988	-0.06468543614987
N	0.91984259536033	-0.14007232370942	-0.09922321901863
H	5.50125782515210	-2.39872635950170	-0.47418989444896
H	6.04757638520652	-1.51855130972632	0.96582606050730

H	6.48739514505481	-0.93533930856891	-0.65170159771915
H	2.79609023454107	2.22338970433562	-0.08322444513971
O	0.55900103538533	-1.29728280121676	-0.10955488568605
O	0.24409214179672	0.85501481606413	-0.09486833293358

[TS analogue]

$E_{ZPE} = -470.6784$ Hartree

C	2.55483900982696	-0.17094077745249	-0.10459315090953
N	3.48770985612687	-1.18028024630405	-0.12439652322122
C	4.64131637412736	-0.59112360099745	-0.06724418085315
N	4.47690088250933	0.79023647109717	-0.01025679908131
H	5.22228362746220	1.47125514383786	0.04003508232948
C	3.17297433845357	1.07517368336230	-0.03261879205280
H	0.66081623116420	-2.17766879780076	-0.24224585268469
C	5.97616468490132	-1.24161917029065	-0.06049140887543
N	1.14003234571755	-0.20661605370249	-0.14430229586371
H	5.84500065494731	-2.32043366921617	-0.11267855395730
H	6.52150063555533	-0.99205665985914	0.85309391737179
H	6.56926501750071	-0.91216893252423	-0.91721806470637
H	2.77291302476434	2.07672294442939	0.00116323999198
O	0.31351780474140	-1.26470764467969	-0.21257610707998
O	0.54198273720129	0.81381774910042	-0.11616077440773

[ring opening]

$E_{ZPE} = -470.4927$ Hartree

C	2.25110333159282	-0.69277432418199	-0.39828063272812
N	4.61516342089347	-1.79152081845237	-0.92024561652571
C	5.02033936885417	-0.79500464720139	-0.28159413591640
N	4.29129390727464	0.22476675803874	0.29773462297159
H	4.80341431394287	0.95046779980954	0.77750776537199
C	2.92711491061991	0.27612297099624	0.24075779502042
H	-0.85679491925061	0.44789269643239	0.10088075763209
C	6.54095103288153	-0.76142717592487	-0.16128994984980
N	0.81939244623171	-0.61549644751008	-0.44452607209002
H	6.98781880394249	-1.62021355913398	-0.65924899397879
H	6.79423836552831	-0.78479825767834	0.90066707343396
H	6.89453075578734	0.15724947286187	-0.63400139569507
H	2.48820008843458	1.13596945620251	0.74041435386983
O	0.16672998635944	0.45857956548356	0.17473510006826
O	0.15372141190671	-1.42022305074178	-0.98400093558421

[Methyl Transference]

$E_{ZPE} = -470.5795$ Hartree

C	2.11686506456687	-0.23602164218730	-0.11461883669439
N	5.71721072370550	-0.53999385421285	-0.06741375503965
C	4.91223552265976	0.25641650417621	0.11008285187408
N	4.01543936708796	1.17499964061544	0.30083418077777
H	4.33514771122665	2.08013351834345	0.62058065136448
C	2.60511371127377	0.96003561638718	0.07722827338258
H	-0.73774726923606	-1.22204803256202	0.27656222507299
C	6.70835574119334	-1.55996103338830	-0.28044735543567

N	0.86509446221344	-0.57379500190949	-0.41087212587554
H	6.18624238463983	-2.45444831493780	-0.62047634481179
H	7.21895179239186	-1.75461309807990	0.66167895712976
H	7.40589357736606	-1.21640389592582	-1.04297568889873
H	2.05276654250565	1.89460221595942	0.07733975890465
O	0.10603179722527	-0.91975076194794	0.66727440364077
O	0.38961609617938	-0.71956142133021	-1.50526745939129

8 Kinematic Considerations for HCD Fragmentation

To confirm the multiple-collision regime of the dissociation process within the HCD cell, we calculated the maximum energy transferable in a single collision. The Thermo Q Exactive system scales the absolute laboratory-frame collision energy (E_{lab}) linearly with the precursor mass-to-charge ratio (m/z).[S1] Using $m/z = 500$ as the reference, the applied collision energy is calculated according to Equation S1:

$$E_{lab} = \text{NCE} \times \frac{m/z}{500} \times z \tag{1}$$

Where z is the charge factor (1 for singly charged positive ions). For the protonated nitroimidazole isomers ($m/z \approx 128$) analyzed at a Normalized Collision Energy (NCE) of 35, the applied laboratory energy is:

$$E_{lab} = 35 \times \frac{128}{500} \times 1 = 8.96 \text{ eV} \tag{2}$$

However, in a single collision event, only a fraction of this laboratory kinetic energy is available for internal conversion and subsequent fragmentation. This maximum available energy is the center-of-mass energy (E_{COM}), determined by the mass of the precursor ion

(m_{ion} = 128 Da) and the mass of the neutral target gas (nitrogen, m_{gas} = 28 Da), given by Equation S2:

$$E_{COM} = E_{lab} \times \left(\frac{m_{gas}}{m_{ion} + m_{gas}} \right) \tag{3}$$

Substituting our experimental parameters:

$$E_{COM} = 8.96 \text{ eV} \times \left(\frac{28}{128 + 28} \right) \approx 1.6 \text{ eV} \tag{4}$$

The calculated maximum internal energy deposited per single collision ($\approx$ 1.6 eV) is significantly lower than the theoretically determined dissociation barriers for the primary fragmentation channels (e.g., $>$ 3 eV for the NO loss). This kinematic limitation demonstrates that the observed fragmentation cannot proceed via a single high-energy impact. Instead, the dissociation must occur through the sequential accumulation of internal vibrational energy over consecutive collisions with the N_2 bath gas. This is strictly consistent with the established collision frequency (e.g., > 6000 s^{-1}) for precursor ions within the HCD cell operating at approximately 10^{-4} mbar,[S2] confirming a multiple-collision regime.

9 Infra-red spectra for $[1M5NI{+}H]^+$ and $[2M4NI{+}H]^+$

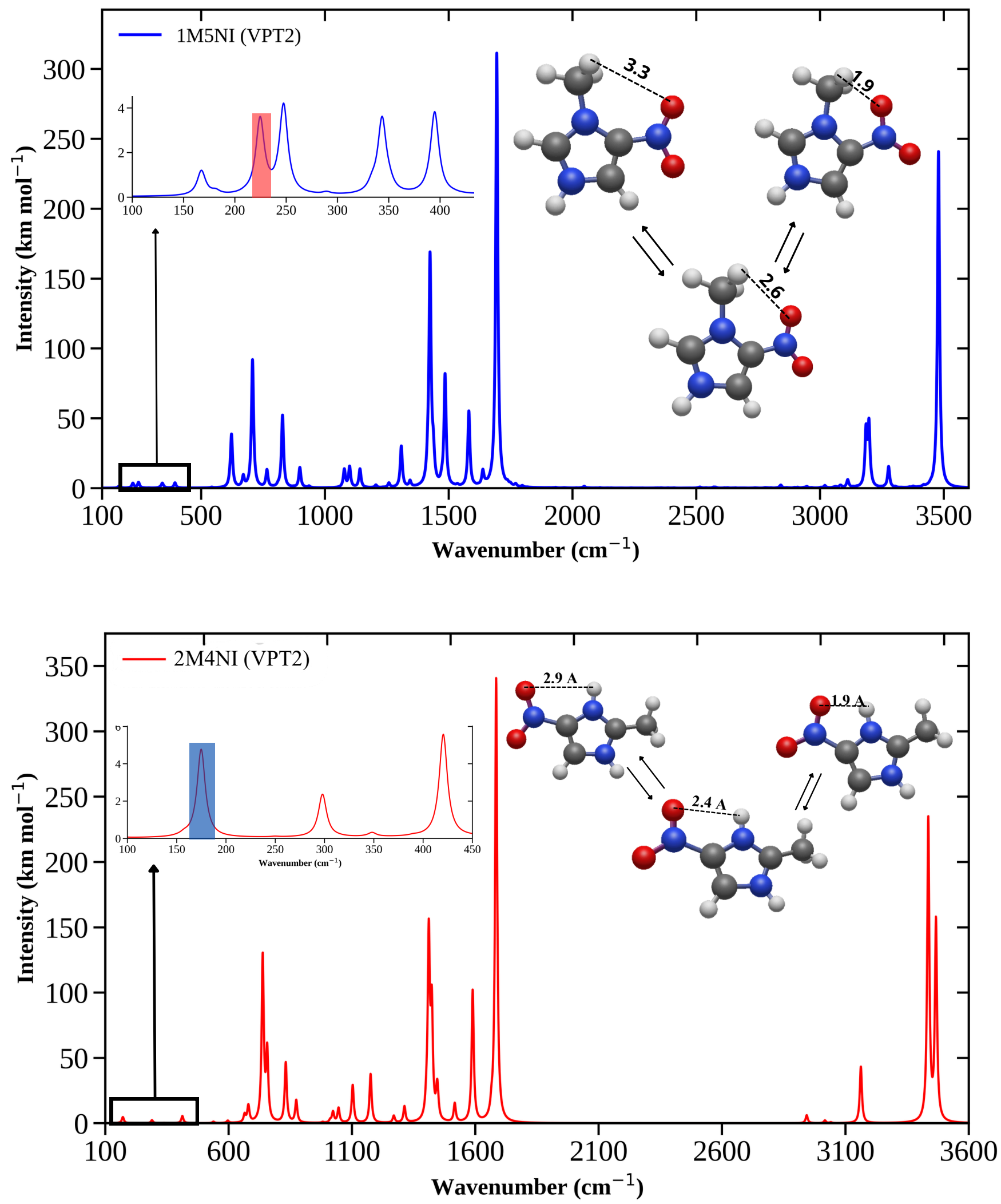

Figure S5: Calculated infrared (IR) spectra for the protonated species [1M5NI+H]$^+$ (**Upper panel**) and [2M4NI+H]$^+$ (**Lower panel**). The inset highlights the in-plane bending vibrational mode region (δ C–N–O), showing the fundamental peaks at 225 cm^{-1} for 1M5NI and 175 cm^{-1} for 2M4(5)NI. The molecular structures depicted in the insets explicitly illustrate the normal mode displacement vectors as a sequence of structural snapshots, demonstrating the dynamic variation in intramolecular distances across a vibrational period. The spectra were obtained including second-order anharmonic corrections (VPT2) at the ωB97X-D4/def2-TZVP level of theory.

References

(S1) Asakawa, D. Phenyl Sulfate Derivatives: New Thermometer Ions for Characterization of Internal Energy of Negative Ions Produced by Electrospray Ionization. *J. Am. Soc. Mass Spectrom.* **2023**, *34*, 435–440.

(S2) Halim, M. A.; Girod, M.; MacAleese, L.; Lemoine, J.; Antoine, R.; Dugourd, P. Combined Infrared Multiphoton Dissociation with Ultraviolet Photodissociation for Ubiquitin Characterization. *J. Am. Soc. Mass Spectrom.* **2016**, *27*, 1435–1442.
